# Supplementary figures and images for: Characterizing the actin-binding ability of Zasp52 and its contribution to myofibril assembly
Source: PLoS One. 2020 Jul 2;15(7):e0232137. doi: 10.1371/journal.pone.0232137 (PMC7332060; doi:10.1371/journal.pone.0232137)

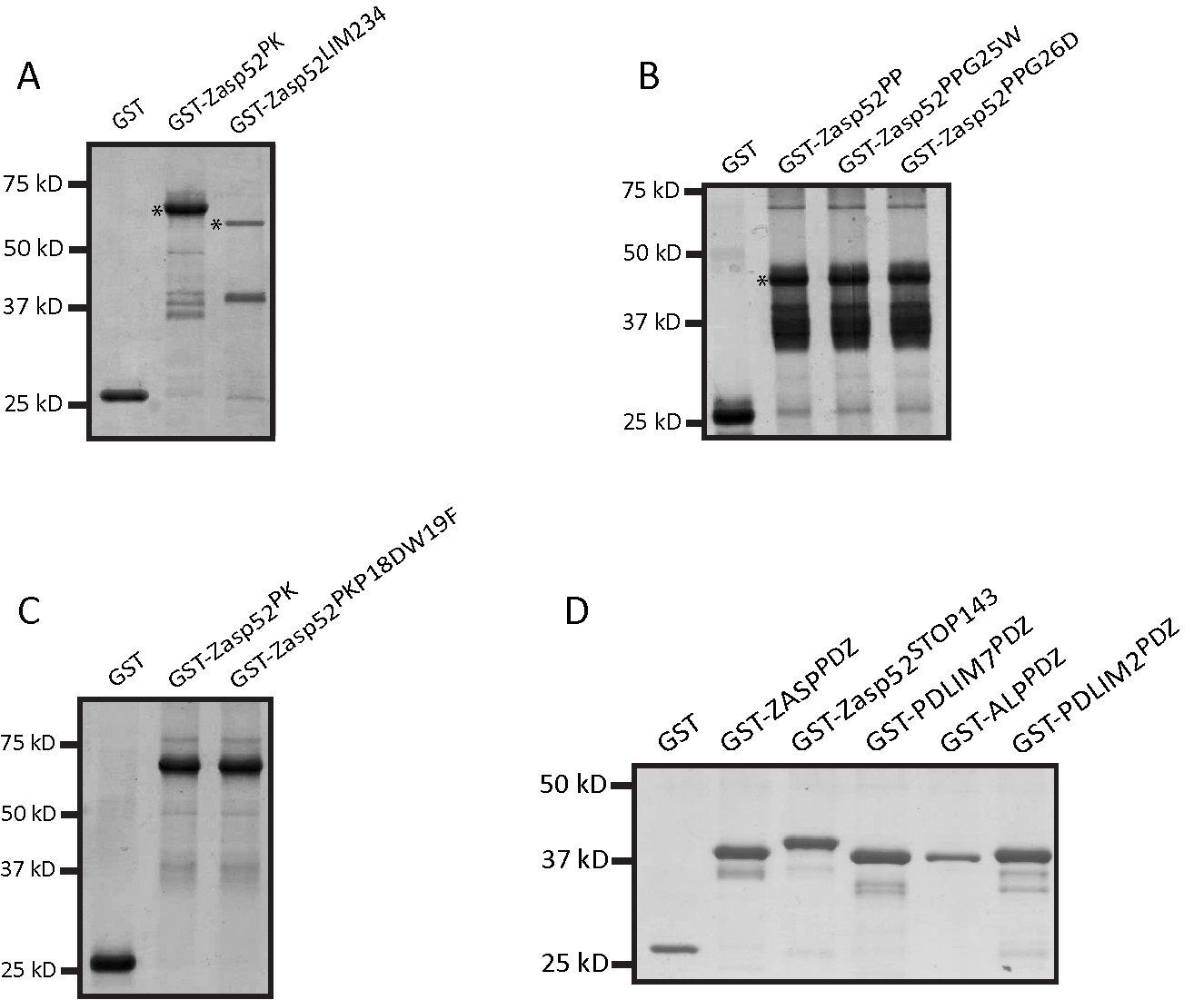

Supplement: S1 Fig — Coomassie staining of Zasp52 domain GST fusions run on a SDS-PAGE gel after purification. (A) GST, Zasp52-PK and Zasp52-LIM234. Asterisks indicate fusion proteins. (B) GST, Zasp52-PP, Zasp52-PPG25W, and Zasp52-PPG26D PDZ domain mutants. Asterisk indicates fusion proteins. (C) GST, Zasp52-PK and Zasp52-PKP18DW19F PDZ domain mutant. (D) GST, human ZASP-PDZ, Zasp52-STOP143, human PDLIM7-PDZ, human ALP-PDZ, and human PDLIM2-PDZ. Molecular weight marker is indicated in kD. (TIF) [file pone.0232137.s001.tif]

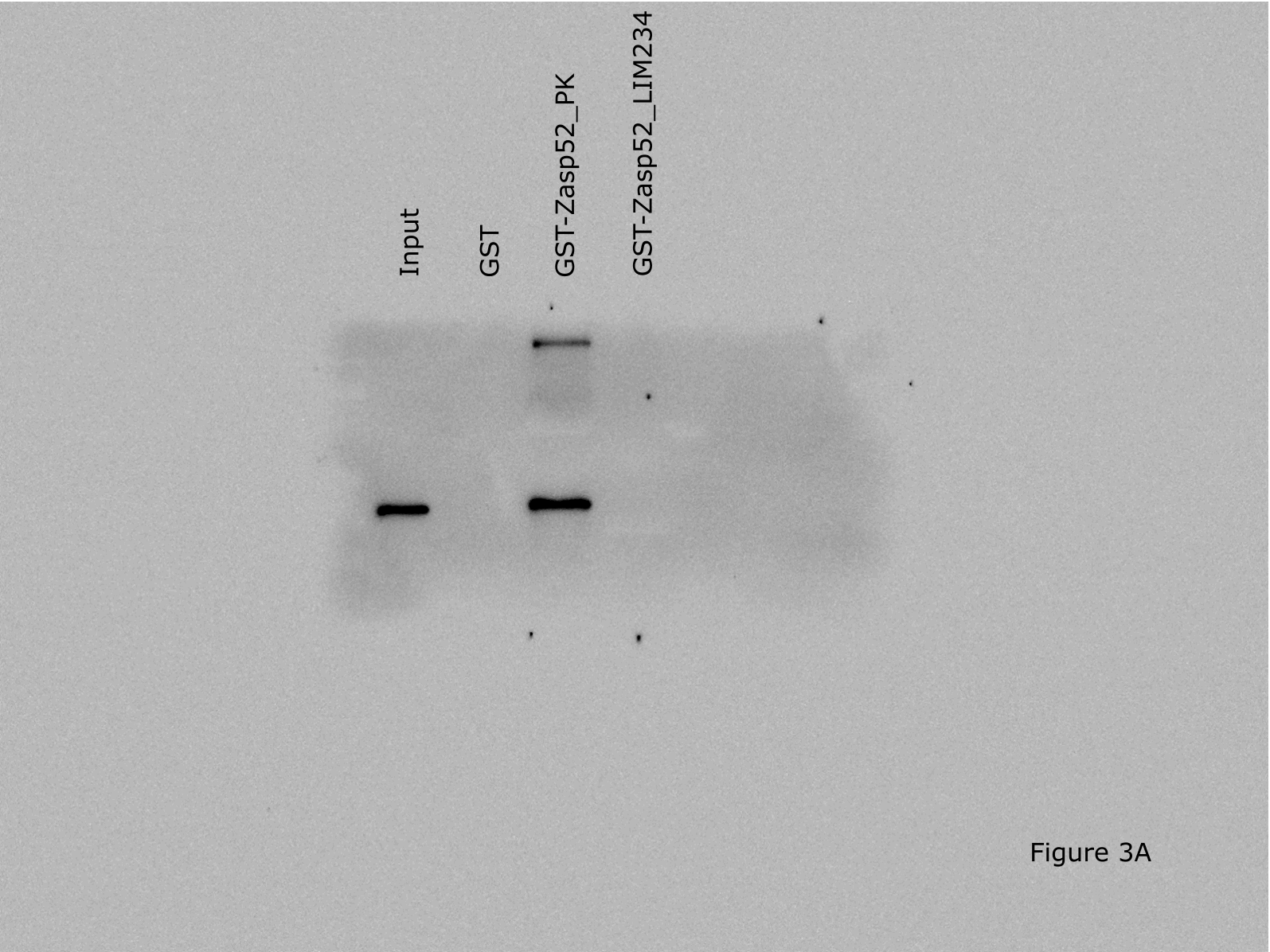

Supplement: S2 Fig — (TIF) [file pone.0232137.s002.tif]

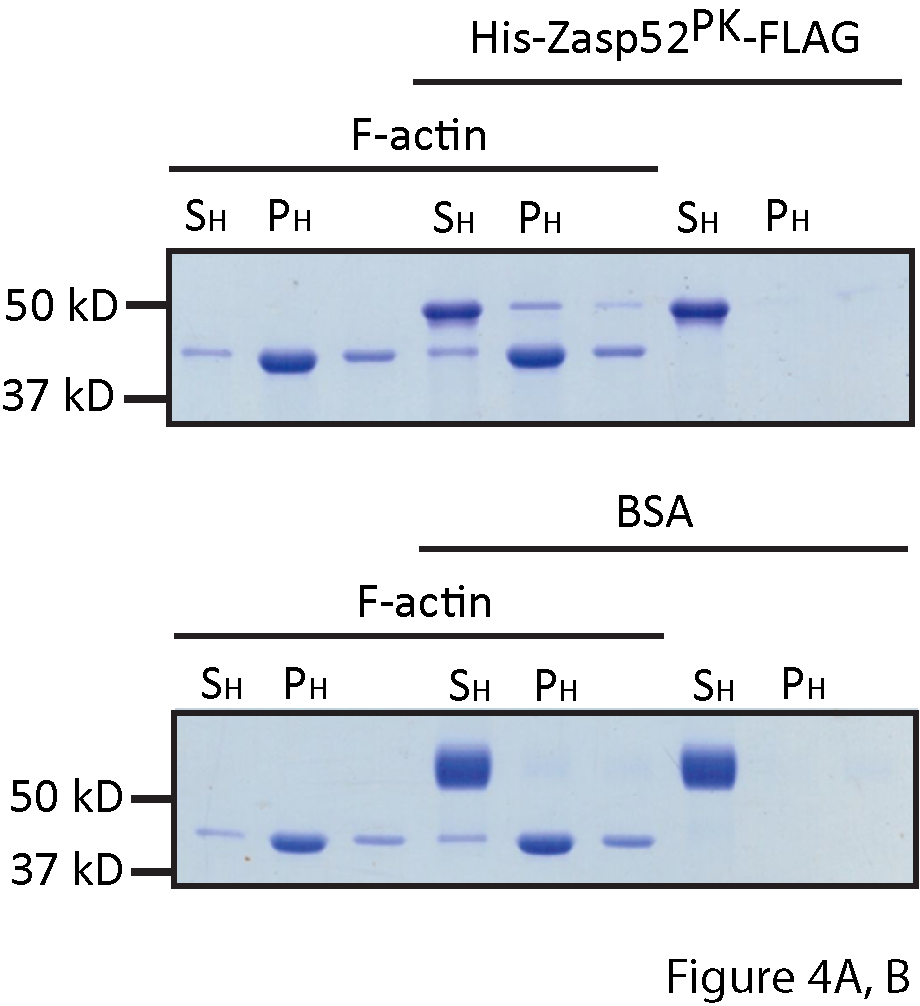

Supplement: S3 Fig — (TIF) [file pone.0232137.s003.tif]

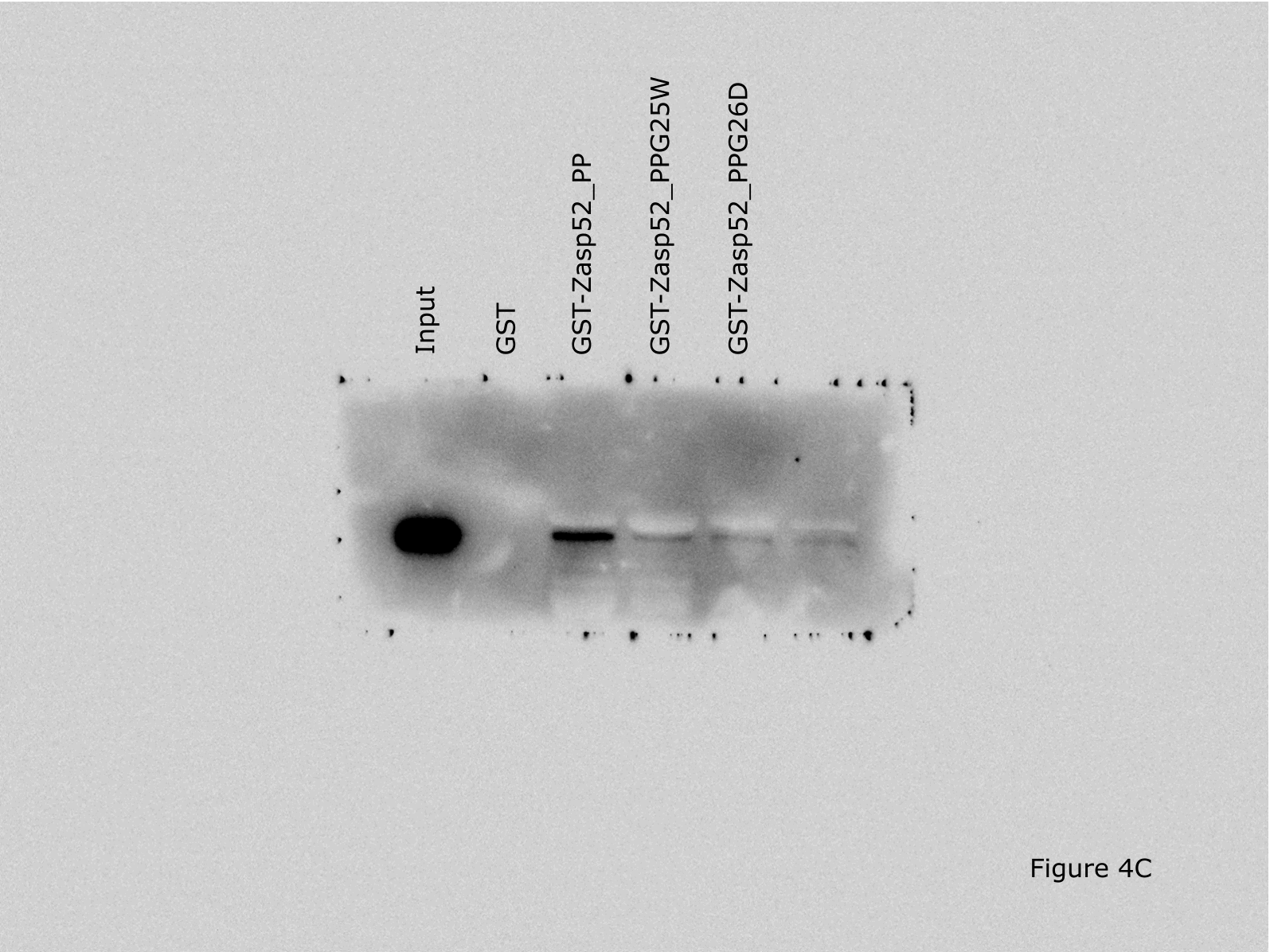

Supplement: S4 Fig — (TIF) [file pone.0232137.s004.tif]

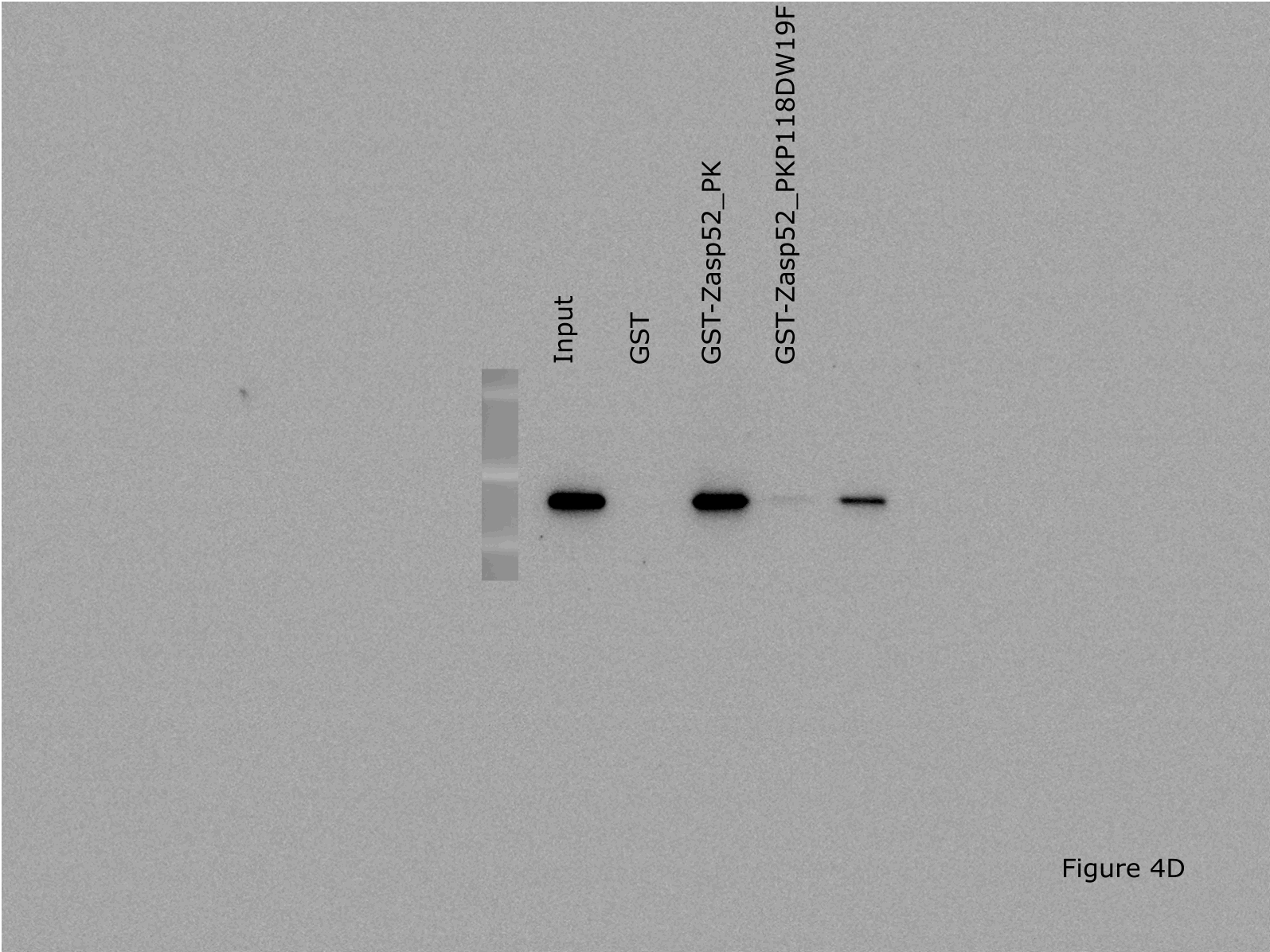

Supplement: S5 Fig — (TIF) [file pone.0232137.s005.tif]

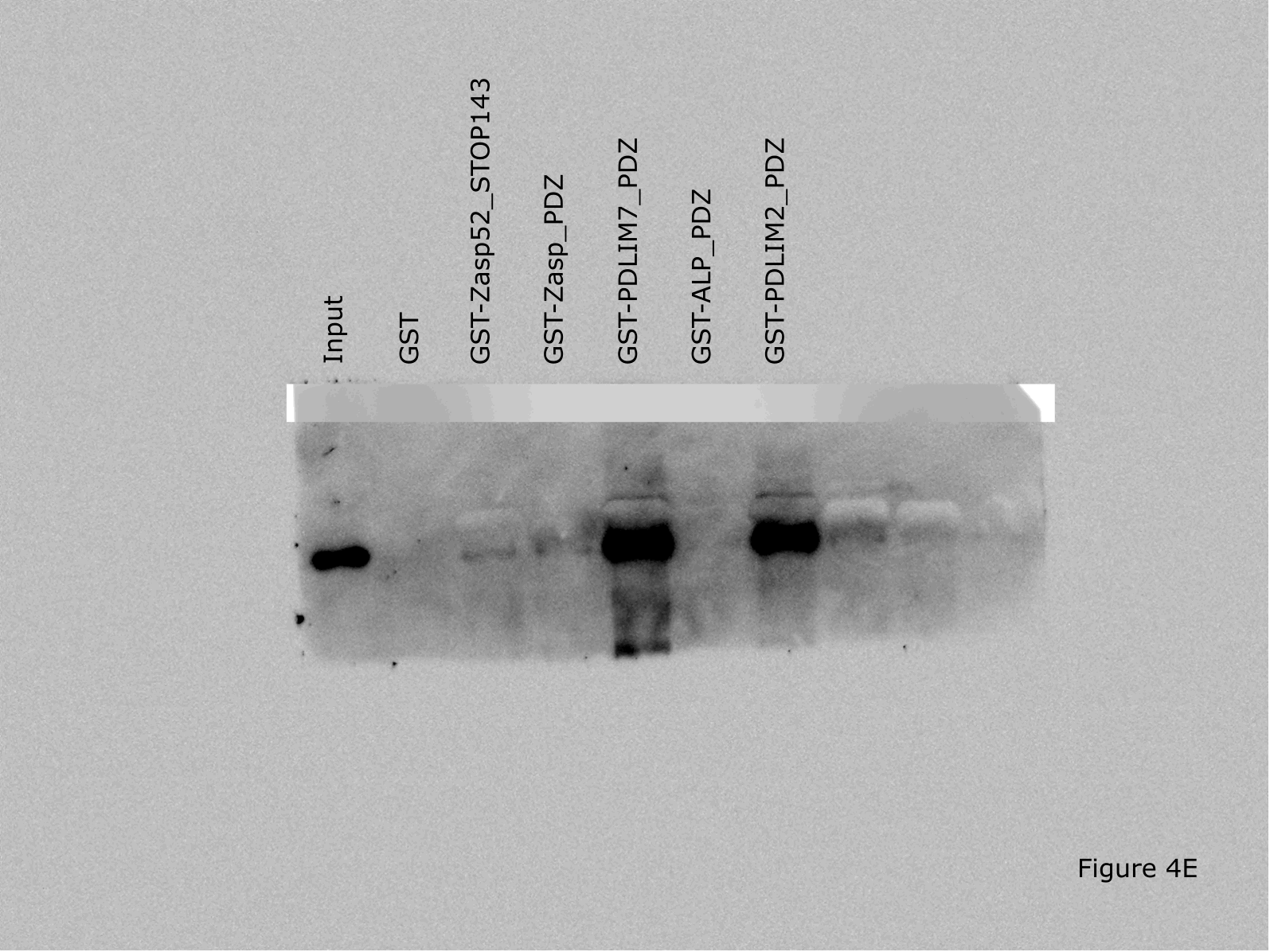

Supplement: S6 Fig — (TIF) [file pone.0232137.s006.tif]

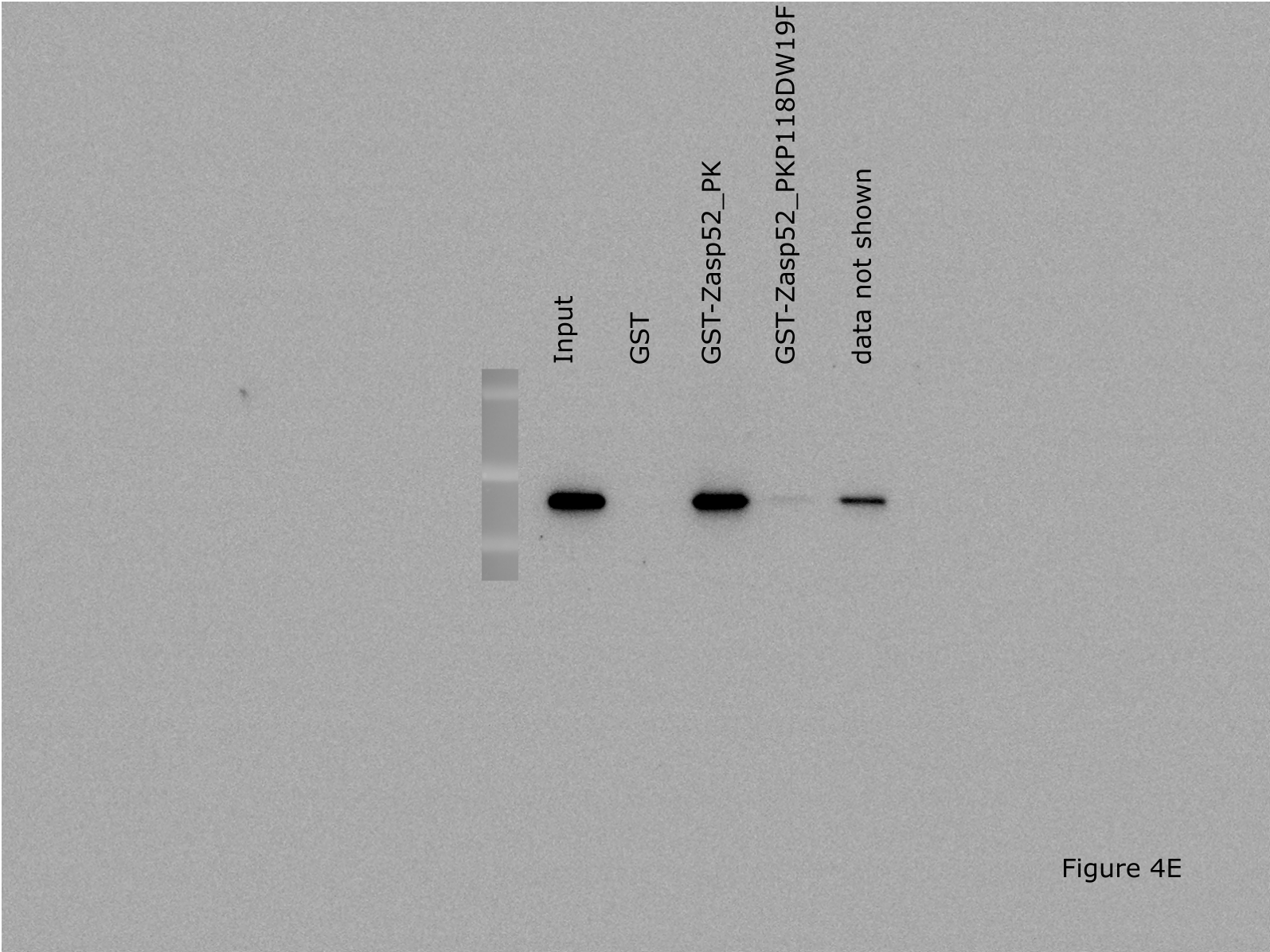

Supplement: S7 Fig — (TIF) [file pone.0232137.s007.tif]

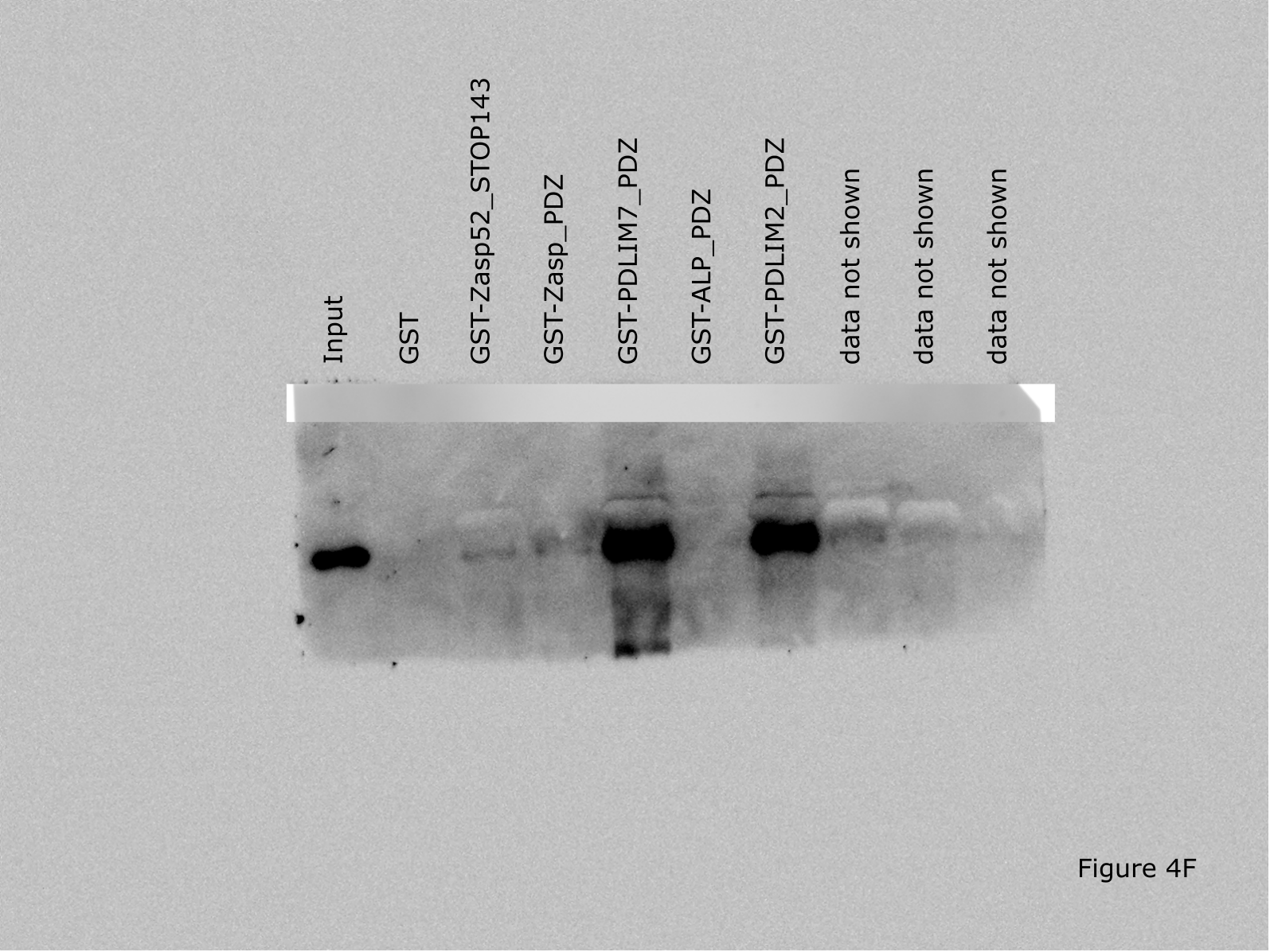

Supplement: S8 Fig — (TIF) [file pone.0232137.s008.tif]
